# The Future Orientation of Past Memory: The Role of BA 10 in Prospective and Retrospective Retrieval Modes

**DOI:** 10.3389/fnhum.2015.00668

**Published:** 2015-12-24

**Authors:** Adam G. Underwood, Melissa J. Guynn, Anna-Lisa Cohen

**Affiliations:** ^1^Department of Psychology, New Mexico State UniversityLas Cruces, NM, USA; ^2^Department of Psychology, Yeshiva UniversityNew York, NY, USA

**Keywords:** prospective memory, retrospective memory, retrieval mode, frontal lobes, Brodmann area 10

## Abstract

Klein made the provocative suggestion that the purpose of human episodic memory is to enable individuals to plan and prepare for the future. In other words, although episodic (retrospective) memory is about the past, it is not actually for the past; it is for the future. Within this focus, a natural subject for investigation is prospective memory, or memory to do things in the future. An important theoretical construct in the fields of both retrospective memory and prospective memory is that of a retrieval mode, or a neurocognitive set or readiness to treat environmental stimuli as potential retrieval cues. This construct was originally introduced in a theory of episodic (retrospective) memory and has more recently been invoked in a theory of how some prospective memory tasks are accomplished. To our knowledge, this construct has not been explicitly compared between the two literatures, and thus this is the purpose of the present article. Although we address the behavioral evidence for each construct, our primary goal is to assess the extent to which each retrieval mode appears to rely on a common neural region. Our review highlights the fact that a particular area of prefrontal cortex (BA 10) appears to play an important role in both retrospective and prospective retrieval modes. We suggest, based on this evidence and these ideas, that prospective memory research could profit from more active exploration of the relevance of theoretical constructs from the retrospective memory literature.

Today I know that such memories are the key not to the past, but to the future

*—Corrie ten Boom*

## Introduction

The purpose of this article is to consider evidence that a retrieval process that supports retrospective memory (i.e., retrieval mode) is also engaged when monitoring is used to accomplish a prospective memory task. In doing so, we hope to encourage researchers in prospective memory to borrow from established theories in the retrospective memory literature to potentially lead to a greater understanding of the processes that support prospective memory. There is a rich history wherein theories from retrospective memory have stimulated thinking about prospective memory. Notable examples include McDaniel and Einstein’s (2007) ideas about spontaneous retrieval (Einstein et al., 2005), which originate in Semon’s and Tulving’s concept of ecphory (Semon, 1921; Tulving, 1983), and Marsh et al.’s (2006) ideas about context effects, which borrow from the concept of encoding specificity. Although this is not an exhaustive list of contributions, there is nonetheless criticism (that we believe may be justified) that prospective memory research could benefit from more exploration of relevant theoretical ideas from the retrospective memory literature (Greene, 2008).

Although this article is not intended to be a comprehensive review of all the evidence that retrospective memory processes are employed during future-oriented memory,^1^ we use a specific process of memory (i.e., retrieval mode) to highlight how theories from retrospective memory might provide insights into how prospective memory operates (Tulving, 1983; Craik et al., 1996; Guynn, 2003). Furthermore, we hope to demonstrate that not only is the construct of retrospective retrieval mode important to understanding prospective memory retrieval mode, but also that these constructs produce similar behavioral effects and rely on the same brain regions (i.e., Brodmann area 10 [BA 10]; see Lepage et al., 2000; Burgess et al., 2011). Moreover, it is also our goal to provide evidence suggesting that prospective memory should not be viewed as a different type of memory than retrospective memory, but as simply basic memory processes employed for future-oriented tasks.

The structure of the paper is as follows: We present the early functionalist arguments that retrospective memory is a system evolved primarily to increase adaptive fitness (see Nairne and Pandeirada, 2008, 2010b) and current functionalist arguments that retrospective memory is for the future and not the past and should thus be studied as a future-oriented system (see Klein et al., 2011; Klein, 2013b). Based on this latter argument, we propose that prospective memory is an ideal candidate to use to evaluate whether retrospective memory theories and neural substrates seem to operate in future-oriented tasks. Moreover, we argue that prospective memory research could be more informative about the nature of memory in general if this approach is adopted. To highlight this view, we review the literature on a specific retrieval process, retrieval mode, which is posited to support both retrospective memory and prospective memory. The review covers the theoretical, behavioral, and neurological (from functional neuroimaging) evidence for both the retrospective and the prospective retrieval mode constructs, with the goal of exploring whether they rely on the same cognitive processes and neural substrates.

## Evolution and Human Memory

There is a recent increase in interest within the mainstream memory literature in the adaptive value or evolutionary significance of human memory.^2^ Researchers seem to be increasingly adopting the perspective that a functionalist approach is likely to yield good insights into the structures and processes of human memory (e.g., Klein et al., 2002, 2010; Addis et al., 2007; Nairne and Pandeirada, 2010b; Nairne, 2010; Schacter et al., 2012). These researchers are exploring the idea, and the evidence for it, that our memory faculties arose as a consequence of evolution by natural selection (e.g., Butler et al., 2009; Tse and Altarriba, 2010; Burns et al., 2011).

Perhaps the most influential recent work has been conducted by Nairne et al. (2007, 2008) and Nairne and Pandeirada (2008; 2010a), who embarked on an interesting series of studies that motivated much subsequent work. They reasoned that if our memories reflect the operation of our brains that evolved for purposes of survival and reproduction in the ancestral environment, then this could be evidenced in the results of straightforward laboratory experiments. If these ideas have merit, then one might expect to find, for example, a mnemonic advantage for stimuli that are particularly relevant to survival and/or reproduction, compared to stimuli that are not relevant in this regard. Similarly, one might expect to find a mnemonic advantage for stimuli that are processed in terms of their relevance to survival and/or reproduction, compared to stimuli that are not. The latter is the approach they took.

Nairne et al. (2007, 2008) and Nairne and Pandeirada (2008; 2010a) conducted a series of studies in which they compared the mnemonic benefit of survival processing to other types of processing known to produce good memory. Various incidental orienting tasks were used to control the encoding of lists of words. In the critical condition in each study, participants were asked to rate the extent to which the object denoted by each word could be useful in some survival scenario, such as being stranded in the grasslands or in a city of a foreign land. A variety of other tasks known to produce good encoding, including intentional learning, pleasantness rating, self reference, imagery, and generation, were compared to this critical condition. Retrieval was then tested via free recall or recognition, and the typical result was that memory, however measured, was significantly better in the survival-processing condition than in any other condition. This mnemonic advantage for survival processing was taken as evidence that our memories (or perhaps more accurately, our neural structures that enable these memories) evolved in order to support the ultimate evolutionary goal of survival.

This work motivated subsequent efforts by others to try to understand the boundary conditions and to test possible alternate explanations of this robust survival processing effect (e.g., Butler et al., 2009; Tse and Altarriba, 2010; Burns et al., 2011). Perhaps most relevant for present purposes is the work by Klein et al. (2011) and Klein (2013a), who proposed that it might not be survival processing *per se* that produces the mnemonic advantage. Rather, the survival processing benefit may occur because the survival processing task requires participants to consider or plan for the (albeit hypothetical) future, and it is this processing in terms of the future that produces the mnemonic advantage. Klein et al. (2011) tested this interpretation in several experiments by varying whether an incidental encoding task involved processing in terms of survival vs. in terms of planning. The important result was a mnemonic advantage for the conditions that involved planning, regardless of whether survival processing was involved, over the condition that involved survival processing alone, without planning. Accordingly, Klein et al. (2011) and Klein (2013a) preserved the evolutionary argument, but its focus was modified to accommodate the new results. Specifically, our critical memory functions are those that were useful in the ancestral environment and that can be considered to result from evolution by natural selection. Among these most critical memory functions is the ability to remember the past in order to more suitably plan and prepare for the future.

A related line of work involves exploring the evidence for common neural substrates involved in thinking backward vs. forward in time (Addis et al., 2007; Schacter et al., 2012). Both these lines of work could be considered to be at least somewhat at odds with a conventional view of memory, that episodic memory is not only about the past, but also for the past. This is reflected in episodic memory experiments in which participants are asked to remember information that occurred earlier in the study, and that is usually the point of the endeavor, at least from the perspective of the rememberer.^3^ In contrast to this conventional view, an emerging perspective is the idea that the evolutionary value and thus the function of remembering is oriented toward the future. In other words, episodic retrospective memory is a byproduct of a system designed to use information about the past for the future. Along these lines, Klein (2013b) suggested that from an evolutionary perspective, it is not particularly useful (or adaptive) merely to remember the past. Rather, what is adaptive is the fact that this remembered information can then be used to plan or prepare for the future. For example, although it does not necessarily seem advantageous, in and of itself, to remember the location of a food source, there is obvious survival significance to being able to remember that information the next time one is hungry and seeking food.

This interesting perspective on the function of episodic (retrospective) memory (i.e., it is for the future rather than for the past) motivates particular types of research questions. Relevant questions include how (or whether, or to what extent) remembered information from the past is used in planning or preparing for the future, and/or how planning or preparing for the future relies on remembered information from the past. Potentially relevant to this focus are a variety of different types of prospection (Gilbert and Wilson, 2007), including prospective memory.

## Prospective Memory

In the context of the ideas outlined by Klein (2013b) it is logical to consider the prospective memory literature. The term prospective memory is generally used to refer to the variety of processes that support remembering at an appropriate time, in the absence of a direct prompt to remember, that some delayed intended activity must be performed. For example, one might wish to remember not to tell a naughty joke while in the presence of certain individuals. In contrast to prospective memory, retrospective memory refers to the processes that support remembering information from the past or information that was encountered at some previous point in time, often in response to a direct prompt or query to remember. For example, one must remember the content of a joke in order to retell it correctly.

In typical prospective memory scenarios, individuals form an intention at one point in time, with the expectation that it will need to be remembered at some future point (i.e., after a delay) without a prompt or query to remember. It seems plausible that information from the past (e.g., memories of prior occasions on which the individual formed a similar intention, and then failed or succeeded at remembering) could be used at the time of formation of the new intention. The remembered information could be used to form an intention that, at least from the perspective of the rememberer, stands a greater chance of being retrieved at the appropriate future point (i.e., compared to a situation in which the remembered information is not used to form the intention). For instance, prior experience provides information about situations in which prospective memory is likely to be particularly challenging or not, the types of environmental events that serve as good or poor delayed intention retrieval cues, and the general quality of one’s own prospective remembering skills. Such information could be used during the encoding and retrieval of subsequent prospective memory tasks, in that individuals could identify environmental events that could serve as good retrieval cues when expecting to be in a situation where one’s prospective memory skills are likely to be challenged. Following from this analysis, one possibility for future research is an exploration of the extent to which (and the possible variety of ways in which) episodic/retrospective memory is (or could be) used in the service of supporting prospective memory. One basic question is the extent to which retrospective memory processes are involved in instances of prospective memory.

This question is not new in the prospective memory literature. Since the advent of laboratory research on the topic (Loftus, 1971), one area of interest (in both empirical studies and theoretical analysis) is the extent to which retrospective memory plays a role in prospective remembering and/or the extent to which the same processes support retrospective and prospective remembering. This interest has been reflected in both a descriptive analysis of prospective and retrospective memory (e.g., a prospective memory task contains both a retrospective component, which is common to both prospective and retrospective tasks, and a prospective component, which is unique to prospective tasks) and empirical investigation of the effect on prospective memory of particular variables (e.g., attention, delay, importance, context, salience) that are known to affect retrospective memory (e.g., Hicks et al., 2000; Kliegel et al., 2004; Nowinski and Dismukes, 2005; Guynn and McDaniel, 2007; Smith et al., 2007).

In terms of a theoretical analysis, more recently a construct has been borrowed from the retrospective (episodic) memory literature and invoked to try to account for some findings in prospective memory. This construct is that of a retrieval mode (Tulving, 1983). In both literatures, a retrieval mode is an important element or prerequisite for retrieval under at least some conditions. Indeed, in Greene’s (2008) review of the most recent edited book devoted to prospective memory (Kliegel et al., 2008), he suggested that one area where prospective memory researchers were making an obvious effort to relate prospective memory to cognition more generally (and episodic/retrospective memory more particularly) was with the construct of the retrieval mode. The purpose of the remainder of this paper is to compare this construct across the two literatures within the framework of Klein (2013b) proposal concerning the future orientation of past memory. The primary evidence we bring to bear on this comparison comes from functional neuroimaging. We first discuss this construct as it has been introduced and explored in the episodic retrospective memory literature, and then we consider it in the context of more recent work on prospective memory. In each case, we examine the behavioral evidence for the construct and then evidence (where available) from positron emission tomography (PET), event-related potential (ERP), and functional magnetic resonance imaging (fMRI) studies.

## Retrospective Retrieval Mode

Many of the most interesting ideas about episodic (retrospective) memory come from the work of Tulving (1983). An influential theory that continues to guide research (Lepage et al., 2000) posits two processes responsible for episodic memory retrieval. The more important for present purposes is the retrieval mode (sometimes referred to as retrieval set, retrieval effort, or retrieval orientation). This is a construct that was originally introduced as one of two components in a theory of episodic (retrospective) memory. The retrieval mode is a sustained (across multiple trials as opposed to transient or trial-by-trial) neurocognitive set or predilection to treat environmental stimuli as potential cues for retrieval of prior episodes. It enables thinking backward or forward in subjective time (i.e., mental time travel) and is invoked when individuals are given explicit or intentional retrieval instructions (but not necessarily on implicit or incidental retrieval tasks). It also helps the rememberer to avoid distraction and to refrain from task-irrelevant processing. It is responsible for recognizing that (or whether) the retrieved information is the intended target of the retrieval attempt (i.e., whether it is a remembered event). Finally, it “mentally holds in the background of focal attention a segment of one’s personal past” (Lepage et al., 2000, p. 506). We particularly like the idea that the information is held in the background of focal attention, in much the same way that activated long-term memory contents are stored in Cowan’s (1999) model. Finally, a more transient process of ecphory refers to the actual recovery of the stored representation (the engram) from long-term memory and into conscious awareness.

### Behavioral Evidence

Behavioral evidence for the distinction between retrieval mode and ecphory comes from finding that the magnitude of impairment on a concurrent task (indicating effort expended toward retrieval, or retrieval mode) is not correlated with the number of items actually retrieved (indicating the success or failure of the retrieval attempt, or ecphory). As an example, Craik et al. (1996) conducted a set of studies on the effects of dividing attention at encoding vs. at retrieval. Participants performed a concurrent key-pressing task (a divided attention task) while either encoding or retrieving a list of words. Whereas dividing attention at encoding impaired memory for the information, dividing attention at retrieval did not. Just as many encoded words were retrieved while participants performed the key-pressing task at retrieval as when they did not. However, the act of retrieval (or attempting retrieval) produced a large cost. Specifically, performance on the key-pressing task was significantly worse when participants were simultaneously attempting to retrieve from memory than when they were not. The conclusion was that there was some sustained process that was supporting, and in fact was necessary for, retrieval, but that effort toward this process was not actually correlated with or predictive of the number of items retrieved. This cost on the concurrent reaction time task, which did not vary as a function of the number of items retrieved, was considered behavioral evidence for the episodic retrieval mode.

### Evidence from Functional Neuroimaging

Further evidence for an episodic (retrospective) retrieval mode comes from cognitive neuroscience. In fact, functional neuroimaging has been particularly useful in this regard, as behavioral results do not lend themselves well to providing evidence for cognitive processes like retrieval mode that are thought to be sustained over time (Rugg and Wilding, 2000). The basic idea here is to identify the areas of the brain that become more active when individuals attempt to retrieve from episodic memory, regardless of whether the retrieval attempt succeeds. The results of these studies from cognitive neuroscience that are most relevant for present purposes are nicely captured by a multistudy analysis reported by Lepage et al. (2000). The analysis involved data collected from four separate, prior studies from their laboratory (Kapur et al., 1995; Duzel et al., 1999; Nyberg et al., 1995, 2000). All studies used PET and tests of recognition memory. The analysis revealed several brain areas that became significantly more active when participants were considered to be in a retrieval mode. Specifically, there was right and left frontal pole (BA 10) activity, right and left frontal operculum (BA 47/45) activity, right lateral dorsal prefrontal cortex (BA 8/9) activity, and activity in the right anterior cingulate gyrus. This pattern or constellation of activity was associated with the attempt to retrieve the memory regardless of the success or failure of the attempt. The take-home message from this analysis was that BA 10 (encompassing the frontopolar prefrontal cortex, rostrolateral prefrontal cortex, and anterior prefrontal cortex) can be considered the anatomical site for the episodic (retrospective) retrieval mode.

Consistent with this interpretation, Velanova et al. (2003) found evidence for sustained activity in and near BA 10 with a procedure better suited to reveal sustained memory processes. In the context of a mixed blocked/event-related fMRI study, they created a condition designed to require a high degree of cognitive control at the time of retrieval, by having participants encode a list of 60 words with a deep/meaningful (i.e., pleasantness rating) task. In comparison, a condition designed to require a low degree of cognitive control at retrieval involved having participants go through 20 trials of intentionally encoding and retrieving a list of 60 words. The results showed a sustained activation in the right fronto-polar cortex near BA 10 in the high control condition but not in the low control condition (compared to a baseline condition).

## Prospective Retrieval Mode

This retrieval mode construct has been invoked more recently in the prospective memory literature (e.g., Guynn, 2003, 2008) as one of two processes involved when individuals monitor for prospective memory targets, which indicate when it is appropriate to realize a delayed intention. The evidence that participants are monitoring, or engaging in some strategic or resource-demanding process as a means of accomplishing the prospective memory task, comes from a finding of task interference.^4^ The theory invoking a prospective retrieval mode was an attempt to begin to specify more precisely what participants might actually be doing in the service of this monitoring for the prospective memory targets. Two processes were proposed, each of which could be responsible for producing this task interference. One process is a retrieval mode; the other is checking the environment for possible targets. Our initial assumption was that a retrieval mode is a pre-requisite for target checking (although we do not have any direct evidence for this, the focus of this article is the retrieval mode and not the target checking). So, conceivably, there could be instances where an individual is both in a retrieval mode and checking the environment for targets, as well as other instances where an individual is in a retrieval mode and not checking for targets. It remains an open question for the moment (although some data that may be relevant are presented later in this article), as does whether prospective memory targets could be detected when one is in a retrieval mode but not checking the environment.

Similar to the retrospective retrieval mode, the prospective retrieval mode is conceived of as a readiness or predisposition to treat environmental stimuli as potential retrieval cues, and in this case, as potential cues to retrieve an intention. This prospective retrieval mode is thought to demand cognitive resources, much like the retrospective retrieval mode, and thus it is believed to support prospective memory when prospective memory relies on demanding cognitive resources. Thus, the retrieval mode is not conceived of as part of a theory of prospective memory *per se*, but rather as a theory of monitoring, or a theory of prospective memory when prospective memory is supported by attentionally-demanding cognitive processes.^5^ The prospective retrieval mode is thought to help support prospective remembering when sustained attention to the relevant aspects of the intention is required to accomplish the prospective memory task (cf. Einstein et al., 2005). In contrast to target checking, which is thought to entail more transient and bottom-up processes, the retrieval mode processes are thought to operate in a more sustained and top-down manner.

### Behavioral Evidence

Behavioral evidence for a prospective retrieval mode comes from finding task interference when individuals have a prospective memory task but are unlikely to be checking the environment for a target or otherwise engaging in any trial-by-trial or stimulus-by-stimulus process.^6^ One way this is achieved is to signal in some way that a particular trial or stimulus will not feature a target. To the extent that checking is effortful and under voluntary control, it is reasonable to expect that individuals would generally take advantage of this information and refrain from checking for a prospective memory target when signaled that one will not occur. Under these conditions, any task interference that occurs, by process of exclusion, would be presumed to reflect the operation of a retrieval mode, because checking for the target is not necessary. Of note, to the extent that this checking is costly, one would expect to find evidence of this in terms of worse ongoing task performance (i.e., greater task interference) under conditions that promote more checking. But for present purposes, we are only interested in task interference when individuals are unlikely to be checking but nonetheless have a prospective memory task to perform.

In an early study exploring the distinction between retrieval mode and target checking, Guynn’s (2003) aim was to create experimental situations in which different combinations of the two proposed processes would likely be operating, and then to look for predictable effects of these combinations of processes on reaction times on an ongoing task in which the prospective memory task was embedded. To get at this issue, participants performed a short-term memory task in which the prospective memory task was embedded, along with a concurrent key-pressing task. Reaction time on this key-pressing task (i.e., the ongoing activity) was the dependent measure of primary interest. Control and experimental trials either alternated (i.e., control trials and experimental trials occurred on alternate trials) or were blocked by trial type (i.e., there was a block of control trials and a block of experimental trials). Also, each change in trial type was preceded by a signal about the nature of the upcoming trial type, primarily so participants would not forget whether or not they were also supposed to perform the prospective memory task, if a target was detected, on any given trial. The expectation was that the involvement of the retrieval mode and target checking should vary in a predictable way across the experimental trials (i.e., both retrieval mode and target checking), the control trials that alternated with them (i.e., retrieval mode only), and the control trials that were blocked (i.e., neither retrieval mode or target checking) and the demands of these processes were expected to be evidenced by impaired performance on the ongoing activity in which they were embedded.

Both processes (retrieval mode and target checking) were presumed to be engaged on experimental trials (regardless of whether they were blocked or alternated with control trials) because a target could occur on any trial. Neither process was presumed to be engaged on control trials that were presented together in a block, because the target would never occur on any trial. The intriguing prediction concerned the control trials that alternated with the experimental trials. Retrieval mode, but not target checking, was presumed to be engaged on these control trials. The logic was that because the target would not appear on that trial, but could appear on the next trial, retrieval mode would be engaged to prepare to respond on the next trial. Accordingly, the prediction was that response times on the ongoing task should be slowest when both processes were presumed to be engaged, fastest when neither process was presumed to be engaged, and intermediate when one process (retrieval mode) but not the other (target checking) was presumed to be engaged. These were the results that were found. Reaction times were slowest on the experimental trials, fastest on the blocked control trials, and intermediate on the control trials that alternated with experimental trials. From these results, Guynn (2003) concluded that engaging a prospective retrieval mode produces a cost to performance (i.e., task interference; similar to retrospective retrieval mode) because of the cognitive effort required to maintain a readiness to realize the delayed intention. The idea is that the processes of engaging a retrieval mode and checking for targets require cognitive resources that would otherwise be used to perform the ongoing activity, and so when one or both of these processes is operating, performance on the ongoing activity is impaired.

In another similar series of studies (Cohen et al., 2012), participants’ task was to make ongoing lexical (word/nonword) decisions, and to press a key if they ever encountered one of six different target words (word intention) or one of six different target nonwords (nonword intention). Task interference was measured as a decrement in the speed of performing the ongoing (lexical decision) task, compared to the speed of performing the ongoing task in a condition with no embedded prospective memory task. Words and nonwords were randomly intermixed, such that participants could not predict which type of stimulus would appear next. Unequal task interference on the word stimuli and nonword stimuli was found, as a function of whether participants had an intention about words or an intention about nonwords. Specifically, there was significant task interference for word stimuli when participants had an intention about words, and for nonword stimuli when participants had an intention about nonwords. In contrast, compared to the control condition, there was no significant task interference when the ongoing task stimulus did not match the type of stimulus about which the participant had an intention. Cohen et al. (2012) interpreted their results as consistent with the idea of a retrieval mode, albeit a somewhat modified view. That is, results demonstrated that reaction time costs were most pronounced on trials where there was material-specific overlap between intention-related targets and ongoing-task stimuli. Therefore Cohen et al. argued that participants employ a retrieval mode in both the word and nonword contexts; however target checking occurs only in contexts where the prospective memory target and ongoing task stimuli match. Target checking appears to be implemented as more of an online strategy that is applied when the features of the stimuli in the ongoing task match those of the prospective memory target stimuli (see also Cohen et al., 2008; Cohen, 2013).

### Evidence from Functional Neuroimaging

As both the theoretical and behavioral evidence suggest that retrospective and prospective memory may share the common cognitive process of retrieval mode, perhaps not surprisingly, the cognitive neuroscience literature indicates that retrieval mode, whether retrospective or prospective, relies on similar brain structures. During a decade of research on functional neuroimaging of prospective memory, Burgess et al.’s (2000; 2001; 2003) work highlighted rostral prefrontal cortex, approximating BA 10, as a region playing a crucial role in prospective memory when an individual is engaged in monitoring for the intention (i.e., nonfocal prospective memory tasks, e.g., Okuda et al., 1998, 2007; Burgess et al., 2000, 2001, 2003). Neuroimaging investigations typically find that performance of prospective memory tasks, compared with performance of ongoing tasks alone, elicit increased activity in lateral BA 10 and decreased activity in medial BA 10. These results are interpreted to mean that lateral BA 10 plays a role in attending to internally-represented information such as intentions for future action (e.g., retrieval mode). Therefore, the signal in this region is increased during prospective memory performance. In contrast, medial BA 10 is thought to play a role in attending to perceptual information in tasks that can be performed on the basis of well-learned stimulus-response links. Therefore, the signal in this region is increased during performance of ongoing tasks alone (see also Gilbert et al., 2005, 2006a,b).

Consistent with this work, there has been a general consensus (for examples, see Okuda et al., 1998; West et al., 2007, 2011; Cona et al., 2012a,b, 2014; McDaniel et al., 2013) that at least some of the cognitive processes that are involved in performing a prospective memory task are supported by areas in and/or structures of the frontal lobes. This evidence has come from studies involving hemodynamic approaches (PET and fMRI), electrophysiological techniques (EEG and ERP; also MEG), lesion studies, and studies investigating normal older adults with behavioral indices of reduced frontal lobe functioning (McDaniel et al., 1999). A number of studies using functional neuroimaging in particular have produced evidence of selective increased BA 10 activity when participants are in a retrieval mode. That is, this increased activity is found when participants expect a target to appear, regardless of whether one actually does appear, compared to when they expect a target not to appear (and none does). We consider some of these results in the following sections. To anticipate, although all of the methodologies yield data that support provocative conclusions, the most useful data for our query seem to come from fMRI studies that use the mixed block/event-related design (Peterson and Dubis, 2012).

#### PET

The first neuroimaging study on prospective memory involved using PET to explore the role of the prefrontal cortex (Okuda et al., 1998). The study showed increased levels of activation, as evidenced by regional cerebral blood flow in a variety of frontal regions, when participants performed a prospective memory task compared to a control task. Most importantly for present purposes was the finding of increased activity in BA 10 and the right lateral frontal lobe when participants expected that prospective targets could appear during an ongoing activity.

In perhaps the earliest prospective memory study to be interpreted as providing evidence for something like a retrieval mode, (Burgess et al., 2001; see also Burgess et al., 2003) were interested in the brain regions that might be involved in the maintenance of an intention vs. its execution. They used a “cognitive conjunction” design (Price and Friston, 1997; see also Friston et al., 1996) in which each participant performed four different ongoing and prospective memory tasks. The idea here is that one of the challenges for getting reliable and valid information about the brain regions involved in prospective memory comes from the fact that such a great variety of ongoing and prospective memory tasks, along with other methodological differences, makes it difficult to identify brain regions that are uniquely involved in prospective memory. The cognitive conjunction design involves having each participant perform several different ongoing and prospective memory tasks, and then the patterns of activation across all the tasks, in conjunction, are considered. Thus, with this type of design, the patterns of activation that are most likely to be statistically significant are those that are common to all four tasks, and thus unique to prospective memory.

Burgess et al. (2001) compared regional cerebral blood flow as revealed by PET under three conditions. In one, participants performed just an ongoing activity; in another, participants expected that prospective targets could appear during the ongoing activity but they did not (expectation condition); and in another, participants expected that prospective targets could appear during the ongoing activity and they did (execution condition). The critical result concerned the areas of increased activation as revealed by significantly increased regional cerebral blood flow in the expectation and execution conditions on one hand, in which participants were expecting to encounter prospective targets, and in the baseline/control condition on the other, in which they were not. This contrast was intended to reflect any activation differences associated with maintaining (vs. not maintaining) an intention. Significant regional cerebral blood flow increases were seen in several regions, with the important ones for present purposes being the frontal pole (e.g., bilateral activation of BA 10), the right lateral prefrontal cortex, the right inferior parietal cortex, and the precuneus. This was found when participants were expecting prospective targets to occur in the ongoing task, regardless of whether they actually did occur, leading to the conclusion that these particular regions all play a role in maintaining an intention (or prospective memory task) in memory. The most critical result here for our purposes is that the bilateral frontopolar activation was independent of execution of the prospective memory task (indicative of prospective memory task retrieval). It should be noted that the block design used in PET studies biases the interpretation that a particular neural response is sustained as opposed to transient.

#### ERPs

Electrophysiological techniques do not permit the same degree of spatial resolution as the PET (and fMRI) neuroimaging techniques but can provide good temporal resolution of neural activity. It seems plausible that one function of a retrieval mode may be to enable the rapid detection (evidenced by a rapid neural response) of prospective memory target event cues when one is in a retrieval mode. Several studies have used this approach to explore the neural evidence for a retrieval mode. In this section, we briefly review a few select lines of work from different laboratories that used event-related potentials (ERPs) and produced results that were interpreted as consistent with the idea of a prospective retrieval mode.

Bisiacchi and colleagues (Cona et al., 2012a,b, 2014) used this approach productively in a number of investigations of prospective remembering involving younger and older adults, time-based and event-based tasks, and focal and nonfocal target events. In one study, they found a sustained positive modulation of the ERPs on the ongoing task trials when either a time-based or an event-based prospective memory task was added to the ongoing task, compared to a baseline condition where the ongoing task was performed alone (Cona et al., 2012a). This positivity for both tasks began about 180 ms post-stimulus and lasted until about 800 ms post-stimulus. This modulation was found mainly over frontal and prefrontal regions, and it was interpreted as reflecting participants being in a retrieval mode when they had the prospective memory task to remember to perform. The fact that the pattern was similar for both types of prospective memory task was interpreted as evidence that the modulations were reflective of a retrieval mode. An important caveat regarding this and other ERP studies is that because the ERPs are measured at the surface of the scalp, this approach provides poor spatial resolution and thus poor localization of the source of the neural signal. Thus, except for the general conclusion that prefrontal areas seem to play an important role and that the behavioral results might implicate a retrieval mode, it is challenging to identify the responsible brain region or regions (e.g., BA 10) more precisely.

Cona et al. (2012b) compared younger and older adults on a time-based prospective memory task and replicated the important results reported above for younger adults. For younger adults, adding a time-based prospective memory task to an ongoing task produced a phasic positive modulation around 150–300 ms post-stimulus and then another sustained positive modulation around 400 ms post-stimulus that lasted until about 800 ms post-stimulus. Both modulations were distributed across the scalp, as in Cona et al. (2012a), but the later sustained modulation was particularly evident over prefrontal regions. The pattern, considered indicative of participants invoking a retrieval mode, differed for the older adults. The different pattern, both in location on the scalp and in time of occurrence, along with the reduced prospective memory and reduced clock checking, was interpreted as age-related decline in the ability to maintain an intention or retrieval mode.

Cona et al. (2014) compared the neural correlates of event-based prospective memory for focal and nonfocal target events. Target event cues are defined as focal or nonfocal with respect to the ongoing activity in which they are embedded. When the nature of the ongoing activity invites or encourages processing that is relevant to detecting a target event cue, the processing is said to be focal; when it does not, the processing is said to be nonfocal. In the case of this study, the ongoing task was lexical decision (word vs. nonword decision). The focal target cue was a particular word (e.g., daisy), and the nonfocal target cue was a category member (e.g., a flower name). The same general pattern of results, indicative of retrieval mode, was found when either a focal or nonfocal prospective memory task was added to the ongoing task, but the amplitude of the ERP modulations differed between the focality conditions.

Finally, in a study looking at the ERP correlates of monitoring for prospective targets that appeared either rarely or frequently, Czernochowski et al. (2012) found a pattern of neural responses that they also interpreted as corresponding to a prospective retrieval mode. There was a similar sustained positive ERP modulation over frontal and prefrontal cortex when an event-based prospective memory task was added to an ongoing activity. This ERP modulation was not affected by whether the prospective memory targets occurred rarely vs. frequently, and thus it was interpreted as consistent with the idea of a retrieval mode (i.e., a more sustained, rather than transient, retrieval process).

#### fMRI

Simons et al. (2006) used fMRI to investigate cue identification and intention retrieval. A similar pattern of hemodynamic changes was found in the condition where cue identification was challenged and in the condition where intention retrieval was challenged. Specifically, there was increased activation in lateral BA 10 (i.e., evidence of attending to internally-represented information such as intentions for future action) and decreased activation in medial BA 10. Additionally, the increased activation in lateral BA 10 was greater in the difficult intention retrieval condition than in the difficult cue identification condition, but the increased activations were not correlated with reaction times, suggesting that the activation results were not just because of differential task difficulty across the two conditions. Also, in a comparison of uncontaminated and contaminated ongoing trials (uncontaminated trials were the control/ongoing trials that occurred before the instructions about the prospective memory task; contaminated trials were those that occurred after), BA 10 activation was greater for the contaminated trials, even though a prospective memory target never occurred. Thus, the increased BA 10 activation occurred when prospective memory targets were expected, regardless of whether they actually occurred, providing evidence that BA 10 supports maintaining an intention in mind.

Gilbert et al. (2009) were interested in whether there are differences in brain activity as evidenced by fMRI when individuals are given the instruction to do a certain task (a prospective memory task) vs. when individuals are given the option to do a certain task (a task identical to the prospective memory task, but with the goal of maximizing points by succeeding at the prospective memory task as well as the ongoing task). The former (cued) condition should cause participants to form an implementation intention that would allow the environment to trigger remembering. The latter (self-initiated) condition should cause participants to form a goal intention, such that the environment would not likely be sufficient to trigger remembering. There was greater sustained activity in the self-initiated but not in the cued condition. There was also a difference between the two conditions in terms of BA 10 activity. Specifically, there was increased activity in the lateral parts of BA 10 in the self-initiated (goal intention) condition, and increased activity in the medial parts of BA 10 in the cued (implementation intention) condition, on the target trials themselves. This activity was found for the target trials compared to the non-target (ongoing task) trials and thus reflects transient activity. This differential pattern of activity is consistent with the idea that medial BA 10 is responsible for behavior that is triggered by the environment, whereas lateral BA 10 is responsible for behavior that is more stimulus-independent.

Reynolds et al. (2009) also found convincing evidence for the role of BA 10 in a prospective retrieval mode, using fMRI to examine the neural evidence for both sustained (over trials) and transient (stimulus-locked) processes. In particular, they used a hybrid blocked/event-related design, which is ideal for determining whether the increased neural activity because of a prospective memory task is truly sustained over time, rather than a transient increase in activity that occurs on most of the trials (the traditional blocked fMRI and PET designs do not allow this distinction). In the critical condition in their study, a prospective memory task was embedded in an ongoing N-back task. For the N-back task, participants saw a series of words presented one at a time and pressed one key if a word was ever a repeat of the immediately prior word, and a different key if not. For the prospective memory task, participants were asked to press a third key if a word ever appeared in a particular color that had been specified at the beginning of the block of trials. This critical condition was compared to two control conditions; in one, participants performed just the N-back task; in the other, they performed just the color detection task (pressing one key if a word appeared in the specified color and a different key if not). Comparing the critical condition to each control provided information about the activity unique to prospective memory, over and beyond the ongoing activity, and separate from simple target detection. The critical result for our purposes was the areas that showed a sustained increase in neural activity when the prospective memory task was added to the ongoing task. These areas included bilateral anterior prefrontal cortex (BA 10), as well as more posterior prefrontal cortex regions, anterior cingulate cortex, and bilateral parietal cortex. Also revealing was a significant negative correlation between activity in right anterior prefrontal cortex and speed of performing the prospective memory task (but not the simple target detection task). Thus, sustained activity in anterior prefrontal cortex, indicative of a retrieval mode, was associated with faster prospective memory task performance, which would be expected if the retrieval mode plays a functional role in prospective remembering. These areas of activity differed from those associated with increased working memory task demands, also suggesting that the increased activity in anterior prefrontal cortex is indicative of a prospective retrieval mode.

McDaniel et al. (2013) also conducted an fMRI study in which they looked for neural evidence for the role of both spontaneous retrieval (resulting from bottom-up processing) and attentional control (resulting from top-down processing) in prospective memory. They also used the mixed-block/event-related design (see Reynolds et al., 2009) that can be used to distinguish between increased brain activity estimates that are sustained vs. those that are transient. They compared performance on both focal and nonfocal prospective memory targets embedded in a semantic classification ongoing task, where focal targets were words (e.g., table) and nonfocal targets were syllables (e.g., tor). The important result for present purposes is that when the prospective memory task involved nonfocal targets (e.g., targets in which monitoring is needed for good prospective memory performance) sustained neural activity was found in the same prefrontal cortex areas (roughly BA 10) as found in prior work (see Burgess et al., 2000, 2001, 2003). This pattern was not found when the prospective memory task involved focal targets, for which participants would not be expected to be in a retrieval mode.

### Spectral Analysis

Another approach that could profitably be used to explore evidence for a retrieval mode is spectral analysis of signals from electroencephalogram (EEG) or magnetoencephalogram (MEG) recordings. In particular, alpha-band activation seems to reflect a sustained effort to retrieve information from long-term memory (e.g., Klimesch, 2012) and thereby could be a function of a retrieval mode. Such an approach could complement the various approaches utilized in the studies described and potentially provide new insights into sustained efforts at retrieval. The one study that we know about that incorporated this approach is a MEG study in which participants performed a retrospective memory and a prospective memory task (Martin et al., 2006). The goal was to test the possible involvement of supervisory executive processes in prospective memory, which “recruit and maintain attentional resources during monitoring for an environmental marker (environmental event) that signals the appropriateness of performing the PM task … and participate in the initiation of PM task performance” (p. 248). The results showed pronounced alpha activity in frontal regions during the time period between the retrospective memory cue (the word “Memory”) and the retrospective memory target (a particular shape for which participants were supposed to make a memory decision). The results also showed similar pronounced alpha activity in frontal regions following the prospective memory target. Thus the similar activity across the tasks could be interpreted as resulting from a retrieval mode. But because activity prior to the target onset was not recorded in this paradigm, the results are not decisive with regard to a prospective retrieval mode being engaged before (as opposed to after) the appearance of the target. Future work would be well served to investigate this possibility.

## Conclusion

To the extent that similar brain regions are active when individuals have prospective and retrospective memory tasks to perform, in conjunction with converging behavioral evidence that a retrieval mode is implicated in each case, some similarity or relation between the two retrieval modes is suggested. The results from functional neuroimaging studies suggested an important role for BA 10 in the prefrontal cortex. Such similarity or relation can be evaluated in the context of Klein (2013b) proposal regarding the function of episodic memory in planning or preparing for the future (e.g., to accomplish a prospective memory task or realize a delayed intention). The comparison—and the possible finding of a relation—potentially helps to inform ideas about the relation between retrospective and prospective memory, and the retrieval mode construct more specifically. We suggest some ideas along these lines in the final section. Furthermore, it also sheds light on Klein’s (2013b) proposal concerning the future orientation of episodic memory.

## Discussion

We opened this article by discussing some ideas about the adaptive nature of the relationship between uses of memory for the past and for the future. Klein (2013b) proposal was that the functional nature of memory is not about maintaining information about the past for the past, but about maintaining information about the past for the present and the future. Furthermore, Klein argues that if memory is for the future, researchers should study memory accordingly; that is, research on memory should be directed at its future-oriented nature (also see Schacter et al., 2007). Prospective memory is presented as an ideal candidate to study the relationship between retrospective memory and future-oriented memory.^7^Although other processes in prospective memory have borrowed from the retrospective literature (e.g., spontaneous retrieval, context effects), retrieval mode was chosen as our specific vehicle for this topic. A critical reason why we believe retrieval mode is a good mechanism to begin this discussion is because there are years of rich neurological research on this subject in both retrospective and prospective memory (e.g., Lepage et al., 2000; Burgess et al., 2001, 2003; Gilbert et al., 2005).

We presented the theoretical, behavioral, and neurological evidence for retrieval mode, in both retrospective memory and prospective memory, with the purpose of highlighting the shared characteristics. First, the theoretical proposals suggest that retrieval mode processes, in both retrospective and prospective memory, include thinking back in time, avoiding distraction, and recognizing retrieved information as the desired content (e.g., Tulving, 1983; Craik et al., 1996; Guynn, 2003). Theoretically, retrieval mode is maintaining a state of readiness to respond to a memory, whether actively trying to recall an old memory or actively trying to recall an intention. Second, the behavioral evidence suggests that there will be task interference on an ongoing task when retrieval mode is being engaged. That is, maintaining a retrieval mode requires demanding attentional processes that will produce a cost to other cognitively-demanding tasks being performed simultaneously (e.g., Craik et al., 1996; Guynn, 2003). Finally, the functional neuroimaging evidence, whether from PET, ERPs, spectral analysis, or especially fMRI using a mixed block/event-related design, suggests that maintaining a retrieval mode is associated with increased activity in the prefrontal pole, specifically BA 10 (e.g., Lepage et al., 2000; Burgess et al., 2003; Reynolds et al., 2009; Cona et al., 2012a,b; McDaniel et al., 2013). Theoretical proposals for the function of BA 10 suggest that the brain region is responsible for multitasking and maintaining a suspended task while another task is being completed (see Koechlin and Hyafil, 2007; Burgess et al., 2007a,b). With this being the case, BA 10 is a logical region for retrieval mode to occur, whether being utilized retrospectively or prospectively.

An important issue to be addressed is the precise manner in which BA 10 could support a retrieval mode in both prospective and retrospective memory. One possibility comes from the medial/lateral dissociation reported above Gilbert et al., 2009. This finding that being in a prospective retrieval mode was accompanied by increased activity in lateral BA 10 and decreased activity in medial BA 10 has been replicated several times (Gilbert et al., 2005, 2006a; Henseler et al., 2011; see also Gilbert et al., 2006b,c and Uretzky and Gilboa, 2010) and has been interpreted as evidence for a gateway hypothesis of rostral prefrontal function (Burgess et al., 2007a,b). According to this hypothesis, rostral prefrontal cortex is a gateway between two different means of attentional control. One means has been referred to as stimulus-oriented responding (or responding to external stimuli or sensory input from the environment) and is proposed to be supported by the medial areas of BA 10. The other has been referred to as stimulus-independent responding (or responding to internal stimuli in mind or stimuli that are generated or maintained by oneself) and is proposed to be supported by the lateral areas of BA 10. To the extent that this gateway hypothesis continues to receive support in the literature (e.g., Uretzky and Gilboa, 2010; Henseler et al., 2011) and this medial/lateral dissociation can be replicated when individuals are in a retrospective retrieval mode, it could suggest the mechanisms by which BA 10 mediates the retrieval mode for both types of remembering.

In conclusion, after reviewing the memory retrieval process referred to as retrieval mode, we believe this memory mechanism is employed for both retrospective memory and prospective memory tasks. Moreover, we believe the brain regions and cognitive processes that support retrieval mode are similar whether memory is being used for the past or the future. These findings are consistent with Klein (2013b) and others’ (see Schacter et al., 2007) ideas that retrospective memory supports and functions for future-oriented memory. And to better understand retrospective and prospective memory processes, it is prudent to have knowledge about how the memory systems operate and to investigate how these systems function for the future.

Prospective memory is in the position to be at the forefront of investigating these claims. Not only could prospective memory research prosper by considering how retrospective memory processes are used to fulfill intentions, but by doing so, prospective memory could provide new insights into how memory operates in general. As mentioned before, the field has already been employing this technique for some time. Moreover, some of the most well-established ideas and findings in the field of prospective memory (e.g., spontaneous retrieval, context effects) are grounded in retrospective memory theoretical ideas (e.g., ecphory, encoding specify). We believe that being mindful of how retrospective memory processes might support the future-oriented memory processes we are studying can significantly improve our own theories and theories of how all types of memory work.

## Conflict of Interest Statement

The authors declare that the research was conducted in the absence of any commercial or financial relationships that could be construed as a potential conflict of interest.

## Footnotes

^1^For more comprehensive treatment of the subject, see Klein (2013b); Schacter et al. (2007); Schacter et al. (2012) and Szpunar (2010).

^2^As a note, to be fair, Tulving (1972); Tulving (1983); when theorizing about memory systems (i.e., procedural, semantic, and episodic), suggested that memory systems are used for survival and to plan and simulate the future (see also Schacter et al., 2007). But it has been only recently that some memory researchers have fully embraced the idea that memory systems are functionally for the future and should be studied as future-oriented systems (see Klein, 2013b).

^3^From the perspective of the researcher, of course, other goals are possible, including information about the effectiveness of different encoding or retention processes, and the effects of retrieval on subsequent memory processes.

^4^Task interference refers to impaired performance on the ongoing task in which the prospective memory task is embedded, compared to performance on this ongoing task when there is no embedded prospective memory task. Task interference is presumed to occur because individuals are actively trying to accomplish the prospective memory task (e.g., by searching for the target, checking stimuli, and maintaining the intention) while performing the ongoing task.

^5^There may be other occasions when monitoring is not invoked, and prospective remembering is accomplished through more spontaneous retrieval processes, much like the processes of ecphory detailed earlier (e.g., Scullin et al., 2010). The concept of a prospective retrieval mode may not be relevant under these conditions.

^6^As mentioned previously, if monitoring comprises target checking and retrieval mode, then by controlling for target checking, it is possible to observe the behavioral cost of retrieval mode.

^7^Although the paper focuses on the role of prospective memory in investigating how retrospective memory supports future-oriented memory, there have been other influential avenues of research exploring this topic. Research in planning, foresight, and future episodic thought have been investigating the theoretical, behavioral, and neurological relationships among retrospective memory and these future-oriented task for more than a decade (for examples see Addis et al., 2007; Schacter et al., 2007, 2012; Szpunar, 2010).
